# The Correlation Between Thyroid Function, Frontal Gray Matter, and Executive Function in Patients With Major Depressive Disorder

**DOI:** 10.3389/fendo.2021.779693

**Published:** 2021-11-23

**Authors:** Shuai Zhao, Yi Xia, Yinghong Huang, Haowen Zou, Xumiao Wang, Zhilu Chen, Hongliang Zhou, Yinglin Han, Hao Tang, Rui Yan, Zhijian Yao, Qing Lu

**Affiliations:** ^1^ Department of Psychiatry, The Affiliated Brain Hospital of Nanjing Medical University, Nanjing, China; ^2^ Nanjing Brain Hospital, Medical School of Nanjing University, Nanjing, China; ^3^ School of Biological Sciences & Medical Engineering, Southeast University, Nanjing, China; ^4^ Child Development and Learning Science, Key Laboratory of Ministry of Education, Nanjing, China

**Keywords:** thyroid hormones, executive function, gray matter volume, voxel-based morphometry, magnetic resonance imaging, major depressive disorder

## Abstract

The present study was aimed to investigate the relationships between serum thyroid hormones (THs), frontal gray matter volume, and executive function in selected patients with major depressive disorder (MDD). One hundred and four MDD patients and seventy-five healthy controls (HCs) were subjected to thyroid-stimulating hormone (TSH), free Triiodothyronine (fT3), free Thyroxine (fT4), and executive function tests and underwent structural magnetic resonance imaging (MRI). Voxel-based morphometry (VBM) analysis was performed to compare group differences in the gray matter for the frontal lobe. Furthermore, mediation analysis was used to investigate whether gray matter volumes of the frontal gyrus mediated the relationship between serum THs and executive function in MDD patients. MDD patients exhibited significant gray matter volume reduction in several brain regions, including the left rectus, right middle frontal cortex, and left middle frontal cortex. Serum TSH levels are positively associated with altered regional gray matter volume patterns within MFG and executive function. Importantly, gray matter in the right MFG was a significant mediator between serum TSH levels and executive function. These findings expand our understanding of how thyroid function affects brain structure changes and executive function in MDD patients.

## Introduction

Major depressive disorder (MDD) is a common and debilitating illness that affects millions of people worldwide. Recently, cognitive impairment has experienced an increased recognition and has been well-known in general psychiatric practice as a core feature of MDD (1, 2). Cognitive function comprises multiple cognitive domains, such as memory, language, visuoconstruction, perception, attention, and executive functions (3). Depression-related cognitive deficits predict a range of psychosocial outcomes while negatively impacting academic performance, social functioning, and psychosocial development (4). Notably, cognitive impairment often persists after remission of depressive symptoms in 30-50% of patients with MDD (5). A recent study indicated that executive function might be the significant deficit in MDD and likely originated from disease duration’s cumulate effect (6). Executive functions describe various cognitive processes that coordinate, monitor, and maintain other more basic cognitive processes (cognitive flexibility, inhibition, and working memory) involving the frontal lobe steers goal-directed behavior and emotional control (7). Several studies have suggested that the changes in executive function in long-lasting depression showed a state-like phenomenon and were found to be improved by antidepressants and depression recovery (8, 9). However, the currently available antidepressant drugs and behavioral therapies are not always effective in some patients. The reason is probably that the currently available antidepressant medications are mainly designed to target the brain’s serotonergic and/or noradrenergic systems. At the same time, many studies have demonstrated that changes in the neuroendocrine system could impair synaptic plasticity, leading to abnormalities in regional brain activity, including depressed mood and executive function (10).

On the one hand, it is recognized that disturbance in thyroid homeostasis may manifest various psychiatric symptoms, including depression, anxiety, mood swings, and psychosis, probably due to thyroid hormone-mediated interference in metabolic processes and intracellular signaling pathways (11). For example, decreased T4 levels have been linked to reduced neuronal actin polymerization in cultured astrocytes. Furthermore, T3 can limit neurotransmitter release, trigger protein synthesis in mitochondria, and influence gene transportation (12, 13). In addition, abnormalities T4 to T3 ratio blunted TSH response to Thyrotropin-releasing hormone (TRH), and the presence of anti-thyroid antibodies (anti-TPO) are more often observed in depressed patients than in healthy people (14).

On the other hand, THs play an essential role in regulating gliogenesis and neurogenesis from development and aging, and dysregulation of THs has been shown to impact executive function negatively (15, 16). For instance, recent research showed that TSH receptor (TSHR) signaling deficiency-induced cognitive impairment indicated that TSH played a role in cognitive functions (17). A longitudinal study found that low TSH levels were related to poorer performance on executive function tests in middle-aged adults (18). Furthermore, patients with subclinical hypothyroidism (defined as elevated levels of TSH and normal levels of free T4) may experience executive and working memory impairments due to abnormal alterations of brain networks, structural and functional changes (19, 20). THs are crucial for myelination, synaptic plasticity, and receptor function in the central nervous system (21). One may speculate that the relationship between THs and executive function may have a neurobiological basis that remains incompletely understood.

For instance, gray matter volumes reduction in MDD has been well documented in the frontal lobe, mainly responsible for executive function. Previous neuroimaging studies also revealed a consistent relationship between the frontal cortex and executive function in MDD patients. It is important to note that THs may affect frontal gray matter volumes. For instance, a population-based VBM study in Germany suggested that serum fT4 levels were associated with the gray matter volumes in the middle frontal gyrus (MFG) (22). Another study confirmed that thyroid-related gene variants influence regional gray matter volumes in MDD patients (23). Therefore, it is unclear whether serum THs are related to executive function in MDD patients and whether this relationship was mediated by frontal gray matter.

Although the evidence above suggests the crucial roles of serum THs, executive function dysfunction, and gray matter volumes reduction, especially in the frontal lobe, the definite pathophysiology remains indiscernible. In this context, we aimed to investigate the thyroid hormone-related executive function impairment and mediating roles of frontal gray matter in this relationship in MDD patients. The first goal was to examine gray matter volume differences in the frontal gyrus between 104 MDD patients and 75 matched control subjects using VBM because it can provide an objective measure of tissue volume, which is widely used (24). The second goal was to detect the serum THs levels and assess executive function using two cognitive function tests in MDD patients. The third goal was to conduct a mediation analysis to investigate whether gray matter volumes of the frontal gyrus mediated the relationship between THs and executive function in MDD patients. We hypothesized that patients with MDD exhibited lower gray matter volumes in the frontal cortex, more deficits in executive function, and decreased serum THs levels. Furthermore, altered gray matter volumes would mediate the relationship between serum THs and executive function in patients with MDD.

## Methods

### Participants

One hundred four patients (50 males and 54 females) diagnosed with MDD were recruited from the Department of Psychiatry of the Affiliated Brain Hospital of Nanjing Medical University. Inclusion criteria for all participants were aged between 18 and 55 years, being right-handed and Han Chinese. All patients were diagnosed by two experienced psychiatrists using the Structured Clinical Interview for DSM-IV (SCID). Their clinical status was assessed using the 17-item Hamilton Rating Scale for Depression (HAMD) score of > 17 on the day of the MRI scanning. All patients were medication-free for at least two weeks before inclusion. Eligibility criteria excluded patients with any of the following conditions: (1) other Axis I psychiatric disorders, (2) a history of neurologic or organic brain disease, (3) alcohol or drug dependence, (4) pregnancy or breastfeeding; (5) a history of severe physical illness, or MRI contraindications. Meanwhile, seventy five healthy controls (31 males and 44 females) were carefully screened, using the Mini-International Neuropsychiatric Interview (MINI) to rule out a psychiatric illness in patients or first-degree relatives. This study protocol conformed to the ethical guidelines of the Declaration of Helsinki and was approved by the Ethics Committee of the Affiliated Brain Hospital of Nanjing Medical University. All participants signed informed consent forms before participation.

### Clinical and Executive Function Assessment

The clinician for each patient filled out a detailed standardized questionnaire. Additional clinical data were manually retrieved from the patients’ medical records. The HAMD is a 17-item instrument designed to measure the frequency and intensity of depressive symptoms in individuals. A score of 0–7 is considered normal, while a score of 18 or higher indicates at least moderate severity. Here, we used two tests to measure executive function. The Wisconsin Card Sorting Test (WCST) was used to measure global executive functioning: abstract reasoning, strategic planning, organized searching, mental flexibility, and impulse control (25). Participants are asked to classify the stimulus cards correctly according to specific rules. The test allows for the calculation of the number of categories, perseveration errors, and total errors. Perseverative errors were defined as the continued sorting by a category that was no longer correct. Lower raw values (total errors and perseveration errors from the WCST) indicate better performance. In addition, the Backwards Digit Span test was administered to obtain an estimate of working memory (26). In the test, the subjects had to repeat the sequences in reverse order. The primary measures of the test were the digit number length, and the maximum digit number length in the Digit Span Backward was 9. A higher score represents a higher level of function.

### Serum THs Levels Assessments

Peripheral venous blood samples were obtained from all subjects after an overnight fast (8h) within 24 hours following MRI scanning. TSH, free T3 (fT3), and free T4 (fT4) were measured using the electrochemical luminescence method (Roche company Cobas E601 automatic immunoassay). Inter-assay or intra-assay variation coefficients were 5%-9% or 3%-6%, respectively.

### MRI Data Acquisition

The anatomical MRI was acquired using a T1-weighted, three-dimensional, gradient-echo pulse sequence. Subjects were comfortably positioned in a birdcage coil fitted with soft earplugs to reduce head motion and instructed to lie still throughout the scan. The imaging parameters for the T1 mapping MRI were set as follows: repetition time (TR) = 1900 ms, echo time (TE) = 2.48 ms, flip angle (FA) = 9°, 176 axial slices with thickness = 1 mm, in-plane voxel resolution = 1 mm × 1 mm, and field of view (FOV) = 25 × 25 cm^3^. All images were assessed and reviewed for artifacts and structural abnormalities by a qualified neuroradiologist.

### Voxel-Based Morphometry Analysis

Structural images were processed using the VBM method (27) with SPM 8 (Statistical Parametric Mapping, Welcome Department of Cognitive Neurology, University College; London; UK) running under a MATLAB suite (Mathworks, Inc., Natick, Massachusetts). Default settings were used unless otherwise specified. First, image quality was performed by visual inspection. Four participants were excluded from the analysis because of excessive scanner artifacts. Second, for better image registration, set image origin to the anterior commissure. Third, images were segmented into gray matter, white matter, and cerebrospinal fluid using a unified segmentation approach (28). Fourth, the gray matter and white matter files were imported into the Diffeomorphic Anatomical Registration Through Exponentiated Lie (DARTEL) procedure, resulting in resampled tissue class images of all participants (isotropic voxel size of 1.5 mm^3^), which were then warped and modulated to match a temple of average anatomy in MNI (Montreal Neurological Institute) space. Finally, the modulated gray matter images were smoothed with an 8 mm full width at half maximum (FWHM) isotropic Gaussian Kernel. The total intracranial volume (TIV) was calculated by summing the total gray matter, white matter, and cerebrospinal fluid volumes.

### Statistical Analysis

All data analyses were performed using SPSS 19.0. The distribution normality of data was checked using a skewness-kurtosis test. Continuous variables with a normal distribution are presented as the means ± SD, continuous variables with a non-normal distribution as median (interquartile range), and categorical variables were displayed as percentages. Two-sample t-tests, non-parametric tests, or chi-square analyses were utilized to compare the demographic, clinical, THs, and executive function tests groups.

### Between-Group Comparisons of Regional Gray Matter Reduction

We used two-sample t-tests to identify gray matter density changes between the two groups. Age, gender, education, and total gray matter volume were added as covariates of no interest. Multiple comparisons were corrected using the nonstationary cluster-level family-wise error (FWE) method, resulting in a cluster defining threshold of *P* = 0.001 and a corrected cluster significant of *P* < 0.05. According to the previous studies, we explored only within the frontal lobe because it was known *a priori* as the area with executive function in MDD (29, 30). Therefore, the frontal region of interest (ROI) was defined anatomically using the WFU-Pick-Altas implemented in SPM8 (31). The result of the group comparison was used as the inclusion mask in the following mediation analysis.

### Relationship Between Serum THs Levels, Frontal Gray Matter, and Executive Function

ROI-based Pearson correlation analyses were performed to test whether the THs and executive function correlated with brain regions’ gray matter significantly different between groups. Given the exploratory nature of correlational analyses, a significant threshold was set at *P* < 0.05.

### Mediation Analysis

Mediation analysis was conducted to test whether the gray matter volumes served as a neuroanatomical basis for the association between THs and executive function in Hayes PROCESS macro for SPSS (32). PROCESS uses observed variable ordinary least squares path analysis to estimate direct and indirect effects and bootstrapping methods for the confidence intervals; yielding results are minimally affected by sample size. Namely, the total effect of X on Y (c) = indirect effect of X on Y through M (a × b) + direct effect of X on Y (c’). The significance of the indirect effect within mediation analysis was estimated utilizing bootstrap sampling and bias-corrected 95% confidence intervals (CI) based on 5000 bootstrap samples. In the PROCESS analysis, a significant indirect effect was indicated by a percentile bootstrapped corrected 95% confidence interval (95% CI) that does not include zero. In this study, only variables that remained statistically significant associations with others in the correlation analyses were considered independent, dependent, or mediating variables in the mediation analysis. Age, sex, education, TIV, and HAMD scores were adjusted for as confounders. Two-tailed significant levels were set at 0.05.

## Results

### Demographic Results

Demographic and clinical data of all study participants are given in **Table 1**. One hundred and four patients (50 males and 54 females) with MDD and seventy-five HCs (31 males and 44 females) were recruited. No significant differences were found between groups regarding age, sex, years of education, and marriage between groups (all *p* > 0.05). For the MDD patients, the mean HAMD score was 23.4 ± 5.1, and forty-three were recurrence.

**Table 1:** Demographic characteristics, clinical parameters, and thyroid function parameters in MDD patients and healthy controls.

	MDD (n = 104)	HC (n = 75)	t/χ2/*U*	*P*
Demographical and clinical measures				
Age, years	30.5 ± 8.7	31.4 ± 7.8	-0.713	0.477 (t)
Female gender, n (%)	54 (51.9%)	44 (58.7%)	0.800	0.371 (c)
Education, years	14.4 ± 2.7	15.1 ± 2.4	-1.804	0.073 (t)
Married, n (%)	46 (44.2%)	30 (40.0%)	0.319	0.572 (c)
Recurrence, n (%)	43 (41.3%)	–	–	–
HAMD	23.4 ± 5.1	–	–	–
Serum concentrations				
FT3 (pmol/L)	4.72 (4.25-5.57)	4.68 (4.39-5.13)	3803.500	0.778 (u)
FT4 (pmol/L)	15.90 (14.15-18.10)	16.4 (15.6-18.4)	3374.000	0.124 (u)
TSH (mIU/L)	1.61 (1.10-2.16)	2.43 (1.64-3.12)	2088.000	<0.001^*^ (u)
WCST				
Preservative errors	9 (6-14)	7 (5-12)	2899.000	0.002^*^ (u)
Total errors	19 (13-30)	15 (10-22)	3096.000	<0.001^*^ (u)
Categories	5.3 ± 1.6	5.7 ± 0.9	-2.763	0.006^*^ (t)
DSB	5.6 ± 1.8	6.5 ± 1.6	-3.638	<0.001^*^ (t)

MDD, Major Depressive Disorder; HC, Healthy Control; HAMD, Hamilton Rating Scale for Depression; FT3, Free Triiodothyronine; FT4, Free Thyroxine; TSH, Thyroid-stimulating Hormone; WCST, Wisconsin Card Sorting Test; DSB, Digit Span Backward; Comparisons were conducted using t-tests (t), Mann-Whitney U tests (u), and chi-squared tests (c).

^*^P < 0.05.

### Comparisons of Serum TSH Levels and Executive Function

As expected, the MDD patients had lower TSH levels (*U* = 2088.000, *p* < 0.001) compared to the HCs (**Table 1**). No other differences were found to be significant (all *p* > 0.05). Furthermore, the results showed that MDD patients performed worse than controls in all executive function tests (*p* < 0.05).

### Changes in Gray Matter Volumes in MDD Patients

**Figure 1:**
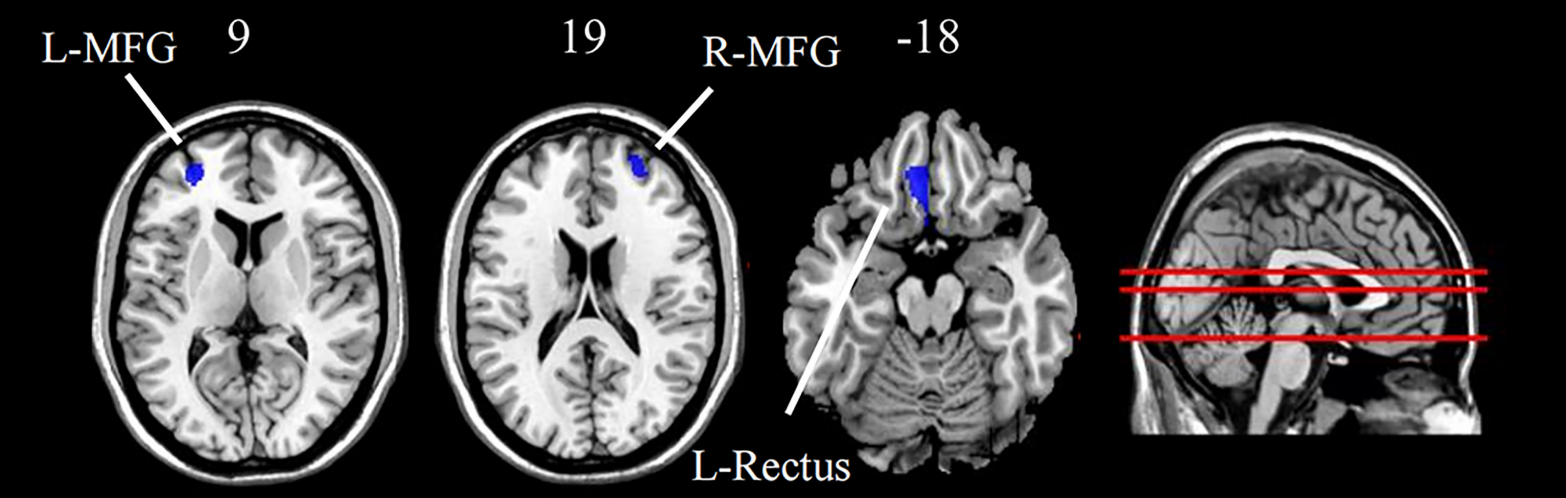
Group difference of grey matter density between MDD patients and HC (FWE cluster-level corrected, p < 0.05). Images are displayed in neurological orientation (left is left) with MNI z axis coordinated. L, left; R, right; L-MFG, left middle frontal gyrus; R-MFG, right middle frontal gyrus; L-Rectus, left rectus.

As shown in **Figure 1** and **Table 2**, compared with HCs, the MDD patients exhibited significant gray matter volumes reduction in several brain regions, including the left rectus, right MFG, and the left MFG (*p* < 0.05, FWE cluster-wise corrected). No regions were identified in which patients had increased gray matter volumes in frontal regions compared to HCs.

**Table 2:** Regional GM volume changes in patients with MDD compared to healthy controls.

Brain region	side	Cluster size	MNI coordinates (mm)			Peak t values	P-value
		(voxels)	x	y	z		(FWE cluster-level)
MDD < HC							
Cluster 1							
Rectus	L	495	-1.5	13.5	-13.5	-4.7473	<0.05
Cluster 2							
Middle frontal	L	553	-28.5	49.5	9	-4.4097	<0.05
Cluster 3							
Middle frontal	R	1305	27	42	33	-4.37	<0.05

x, y, z are the coordinates of primary peak locations in the MNI space; t is the statistical value of peak voxel showing GM volume differences among two groups. P < 0.05, FWE cluster-level corrected.

### Associations Between TSH Levels, Frontal Gray Matter, and Executive Function in MDD Patients

**Figure 2:**
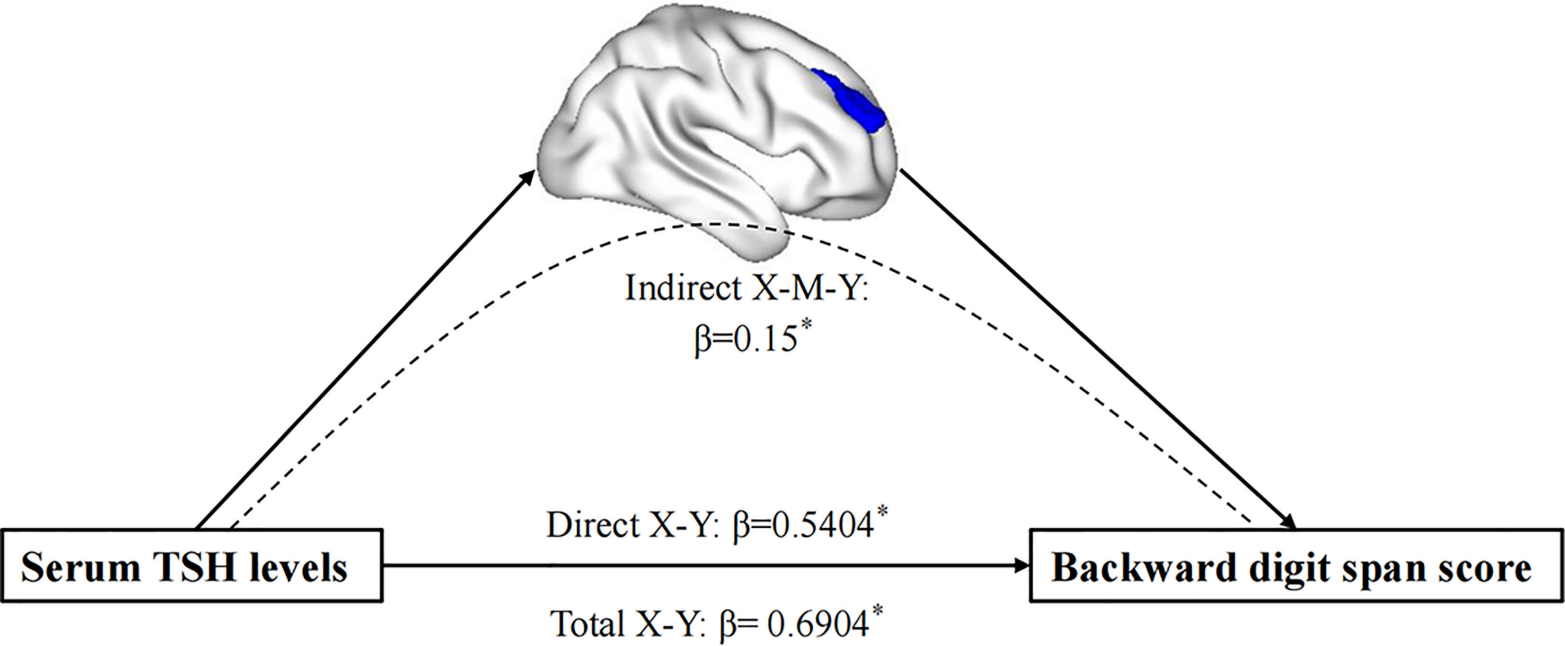
The mediation analysis result. It demonstrates that the GM density of the right MFG mediated the impact of the decreased TSH levels on EF performance in MDD patients. MFG, middle frontal gyrus; EF, executive function. *P < 0.05.

Serum TSH levels were positively correlated with gray matter volumes in the right MFG (*r* = 0.280, *p* < 0.05). The left and right middle frontal gray matter volumes were positively correlated with the backward digit span scores (*r* = 0.383, *r* = 0.223, both *p* < 0.001), and the right middle frontal gray matter volumes were negatively associated with preservative errors (*r* = -0.194, *p* = 0.049). Moreover, serum TSH levels were positively associated with the backward digit span scores (*r* = 0.361, *p* = 0.001) (See **Figure 2**). No association was found between HAMD scores and executive function tests in MDD patients.

### Mediation Analysis

The mediation analysis revealed that the total effect between TSH levels and the backward score showed significant upon the inclusion of gray matter volumes in the right MFG as mediator (path c = 0.690, SE = 0.204, p = 0.0010) (after adjusting for age, sex, education, TIV, and HAMD scores). Bootstrap simulation (n = 5000) further confirmed that the indirect effect of TSH levels on the backward digit span score through gray matter volumes in the right MFG was significant with a 95% CI of [0.0154, 0.3386]. Lower TSH levels with a smaller gray matter concentration in the right MFG predicted a lower backward digit span score in MDD patients.

## Discussion

The purpose of this study was to investigate the effect of THs on executive function in MDD patients and explore whether the gray matter volume changes in the frontal lobe contribute to thyroid hormone-related executive function performance. There were three main findings in the current study. First, MDD patients showed decreased gray matter volumes density in several brain regions, including the left rectus, left MFG, and right MFG. Second, MDD patients demonstrated executive impairment, and the decreased serum TSH levels were positively correlated with executive function in MDD patients. Finally, mediation analysis showed that the decreased gray matter density in the right MFG was a significant mediator between low TSH levels and executive function impairment in MDD patients. These findings suggest that the low serum TSH levels in MDD patients may be related to poor executive function performance, and the decreased gray matter may drive the TSH-related executive impairment in the right MFG.

### Comparison of Serum THs Levels and Executive Function

Consistent with our hypothesis, our results demonstrate that MDD patients had lower TSH levels than HCs, in line with previous reports (33). Although the mechanisms for the association of depression and altered hypothalamic–pituitary–thyroid (HPT) axis activity have not been elucidated, many patients with depression present subtle alterations in thyroid function, as evidenced by the increasing prevalence of subclinical thyroid dysfunction among patients with depression, ranging from 12% to 60% (34, 35). The alterations in thyroid function in MDD might be due to the blunted TSH response to thyrotropin-releasing hormone (TRH), and that the difference between 2300 h and 0800 h TSH responses to TRH (i.e., △△TSH) was reduced in 75% of depressed inpatients (36, 37). In addition, it was hypothesized that the elevation of cortisol levels often found in depression could inhibit the effect of TRH on TSH (36). Another study also suggested that low TSH concentrations might result from a decreased hypothalamic TRH drive (38). Consequently, TRH-induced lower TSH response may be a leading cause of reducing TSH levels, a potential neuroendocrine marker in psychiatric patients (39). Another possible explanation might be that the downregulation of TRH-mRNA in hypothalamic paraventricular neurons resulting from altered hypothalamic deiodinase or MCT8 expression in MDD is a potential cause of lower levels of TSH (38).

### Deficits of Executive Function in Patients With MDD

Executive function refers to higher-order control mechanisms encompassing cognitive flexibility, inhibitory control, and working memory (40). Impairment of executive function is widely recognized as a primary characteristic of MDD (41). In this study, all MDD patients performed worse than controls in all executive tests. First, lower backward digit span scores were found in MDD patients, suggesting that these patients display impaired working memory (42). Consistent with previous studies, completed categories were lower, and total and perseveration errors were higher than in a healthy population (43). These results indicate that MDD patients may exhibit different executive function deficits and have more difficulty adapting their behavior after negative feedback (44).

### Changes in Gray Matter Volumes of Frontal Lobe in Patients With MDD

Our VBM results revealed a significant regional volume reduction in the right MFG, left MFG, and left rectus in MDD patients in the frontal lobe. In addition, no significant differences in structural features were found in other regions. These findings were consistent with previous studies. A meta-analysis found that a lower volume of MFG was commonly detected in MDD patients, suggesting that volumetric alterations in the MFG were specific to MDD (45). In addition, a large-scale VBM study examined gray matter volumetric changes and found that the gyrus rectus was the second-highest regional gray matter volume loss in MDD (46). Although the mechanism is unclear, some studies have found that reductions in neuronal and glial density in the deeper cortical layers of the frontal lobe may underlie the gray matter abnormalities in MDD patients (47).

### The Association Between Thyroid Hormones and Executive Function

It is well established that thyroid hormones are vital for brain maturation, regulating neuronal differentiation and migration, myelination, and synaptogenesis. This study demonstrated the relationship between low serum TSH levels and poorer executive function performance, validated by previous research (18). A previous study indicated that individuals whose TSH levels were slightly decreased within the normal range have an increased risk of Alzheimer’s disease (AD) (48). Other researches also demonstrated that low TSH levels were related to mild cognitive impairment or dementia (49, 50). Nevertheless, some studies also claim the opposite results; higher TSH levels were associated with poor cognitive performance (51) or no association (52). This discrepancy is likely owing to differences in the clinical characteristics and cognitive tests. A possible explanation for our observed results might be the HPT-axis dysfunction in depression. A previous study found that low TRH mRNA expression in patients with MDD might result from a decreased hypothalamic TRH drive (38). Since TRH could increase local acetylcholine synthesis and release in rodents, decreased TRH might also damage the brain’s acetylcholine metabolism (53), which leads to executive dysfunction in patients with MDD.

### Relationships Between Thyroid Function, Frontal Gray Matter, and Executive Function

It is commonly acknowledged that maternal THs play a prominent role in development (54). A recent prospective cohort study found that maternal THs concentrations during pregnancy were associated with lower gray matter volumes, which indicated that THs affect brain development (55). A large UK Biobank study also provided evidence for the effect of THs on gray matter volumes in adults, which extends our understanding of the effect of THs on the neuronal structure in the human brain (56). In our study, serum TSH levels were decreased and positively associated with gray matter volume in the right MFG in MDD patients. Previous studies demonstrated that TSH receptor gene expression was correlated positively with the level of circulating TSH in serum, suggesting that serum TSH may regulate TSH receptor expression in the brain (57, 58).

Moreover, a post-mortem study by Naicker et al. shows that the TSH receptor was expressed in the prefrontal cortex, suggesting that the decreased serum TSH levels might negatively affect the gray matter in the MFG through a TSH receptor-mediated mechanism (59). These results suggest that HPT axis functions are abnormal in MDD patients, negatively influencing gray matter volume in the frontal lobe, especially the MFG, and further influencing executive function.

To the best of our knowledge, few studies have investigated the correlation between THs and gray matter and the potential effect on executive function in patients with MDD. More importantly, results revealed that the altered gray matter density in the right MFG might mediate the serum TSH levels and executive function in MDD patients after adjusting for age, sex, education, and baseline HAMD scores. It is suggested that the MFG is a critical node in the neural circuit underlying the link between serum TSH levels and executive function in patients with MDD. The MFG, a vital component of the dorsal lateral prefrontal cortex (DLPFC), is involved in attention (60), cognitive control functions (61), and working memory (62). Our study replicates previous results showing that greater gray matter volumes in the MFG were related to better executive function (63). A study by Cui et al. identified the phospholysine phosphohistidine inorganic pyrophosphate phosphatase (LHPP), a gene related to thyroid function, was associated with pathological and physiological changes in MDD (64). In another study, the author further found that there were LHPP gene (rs35936514) interaction effects on the dorsolateral prefrontal cortex (DLPFC), suggesting that genotype-disease interaction might have significant effects on morphology in the prefrontal cortex (PFC) in patients with MDD (65). In this context, the results from our VBM analysis may support the hypothesis that altered HPT in MDD patients affects gray matter volumes in the MFG, with a secondary dysfunction of executive function. Although caution should be taken in interpreting these findings, this hypothesis seems to be in line with previous evidence suggesting that a dysfunction of the HPT axis could be associated with frontal lobe gray matter and further contribute to executive function impairment in patients with MDD.

Additionally, we find no association between HAMD scores and executive function tests in MDD patients. The results indicate that executive function impairment represents a core feature of depression that cannot be considered an epiphenomenon entirely secondary to depression severity. A meta-analysis also suggests that executive function deficits may be essential trait-markers for MDD since they persist despite remission of depressive symptoms (66). This study only found relationships between serum TSH levels and brain structure and executive function in patients with MDD. On the contrary, no such association was found in HCs. Our results demonstrate that the observed serum TSH-brain relations are particular in MDD patients.

## Limitations

It is essential to acknowledge the limitations of this study. Firstly, since this study is cross-sectional, it is not possible to infer causality. Secondly, we did not investigate morphologic changes in other brain regions in the current study, nor did we detect other cognitive domains (e.g., attention, memory, or processing speed). Thirdly, we explored only brain structural alterations in MDD patients, and future work should explore changes in brain functional connectivity and networks. Fourthly, the sample of this study was small, and our findings should be verified and extended to more extensive, preferably multi-site patient populations. Finally, although potential confounders were adjusted for, residual confounding cannot be ruled out. Further studies on a larger population with a longer follow-up could provide a longitudinal perspective to confirm the interrelationship between thyroid function, brain structure alterations, and executive function impairment.

## Conclusion

In conclusion, findings from our study point to dysfunction of both the HPT axis and executive function in MDD patients. Furthermore, serum TSH levels are related to altered patterns of regional gray matter volumes within MFG. More importantly, exploratory mediation model testing indicates that gray matter volume in the right MFG was a significant mediator of the associations between serum TSH levels and executive function. These findings expand our understanding of how thyroid function affects brain structure changes and executive function in MDD patients.

## Data Availability Statement

The original contributions presented in the study are included in the article/supplementary material. Further inquiries can be directed to the corresponding authors.

## Ethics Statement

The studies involving human participants were reviewed and approved by Medical Ethics Review Committee of the Affiliated Brain Hospital of Nanjing Medical University. The patients/participants provided their written informed consent to participate in this study.

## Author Contributions

SZ and ZJY conceived of the presented idea. YX, YLH, and QL contributed to the design and implementation of the research. YHH, HWZ, RY, and HT carried out experiments. HLZ, XMW, and ZLC performed the analytic calculations. All authors contributed to the article and approved the submitted version.

## Funding

This study was supported by grants of the National Natural Science Foundation of China (81871066); the Jiangsu Provincial key research and development program (BE2018609, BE2019675); the Jiangsu Provincial Medical Innovation Team of the Project of Invigorating Health Care through Science, Technology and Education (CXTDC2016004); the Key Project supported by Medical Science and Technology development Foundation, Jiangsu Commission of Health (K2019011).

## Conflict of Interest

The authors declare that the research was conducted in the absence of any commercial or financial relationships that could be construed as a potential conflict of interest.

## Publisher’s Note

All claims expressed in this article are solely those of the authors and do not necessarily represent those of their affiliated organizations, or those of the publisher, the editors and the reviewers. Any product that may be evaluated in this article, or claim that may be made by its manufacturer, is not guaranteed or endorsed by the publisher.
